# The ATR-Activation Domain of TopBP1 Is Required for the Suppression of Origin Firing during the S Phase

**DOI:** 10.3390/ijms19082376

**Published:** 2018-08-13

**Authors:** Miiko Sokka, Dennis Koalick, Peter Hemmerich, Juhani E. Syväoja, Helmut Pospiech

**Affiliations:** 1 Department of Biology, University of Eastern Finland, FI-80101 Joensuu, Finland; 2 Institute of Biomedicine, University of Eastern Finland, FI-70211 Kuopio, Finland; juhani.syvaoja@live.com; 3 Leibniz Institute on Aging—Fritz Lipmann Institute, DE-07745 Jena, Germany; dennis.koalick@yahoo.com (D.K.); peter.hemmerich@leibniz-fli.de (P.H.); 4 Faculty of Biochemistry and Molecular Medicine, University of Oulu, FI-90014 Oulu, Finland

**Keywords:** DNA replication, S phase, origin firing, TopBP1, ATR, DNA fiber assay

## Abstract

The mammalian DNA replication program is controlled at two phases, the licensing of potential origins of DNA replication in early gap 1 (G1), and the selective firing of a subset of licenced origins in the synthesis (S) phase. Upon entry into the S phase, serine/threonine-protein kinase ATR (ATR) is required for successful completion of the DNA replication program by limiting unnecessary dormant origin activation. Equally important is its activator, DNA topoisomerase 2-binding protein 1 (TopBP1), which is also required for the initiation of DNA replication after a rise in S-phase kinase levels. However, it is unknown how the ATR activation domain of TopBP1 affects DNA replication dynamics. Using human cells conditionally expressing a TopBP1 mutant deficient for ATR activation, we show that functional TopBP1 is required in suppressing local dormant origin activation. Our results demonstrate a regulatory role for TopBP1 in the local balancing of replication fork firing within the S phase.

## 1. Introduction

The DNA replication program of a mammalian cell is controlled at two distinct phases to guarantee that duplication of the genome occurs once and only once every cell cycle. Early in the gap 1 (G1) phase, all the potential origins of DNA replication are licensed by loading a pre-replication complex consisting of the following core components: a replicative helicase core, composed of DNA replication licensing factor MCM2–7 (MCM2–7), DNA replication factor Cdt1 (Cdt1), and cell division control protein 6 homolog (Cdc6) [1]. The loading of the pre-replication complex is functionally separated from origin firing, which requires elevation of the levels of synthesis (S)-phase-specific cyclin-dependent kinase (S-CDK) and Dbf4-dependent kinase (DDK) at the G1/S border. S-CDKs phosphorylate the key proteins, ATP-dependent DNA helicase Q4 (RecQL4), Treslin, and geminin coiled-coil domain-containing protein 1 (GEMC1), which bind to DNA topoisomerase 2-binding protein (TopBP1) [2,3,4,5]. 1 TopBP1 is subsequently involved in loading Cdc45 to the pre-initiation complex that activates the replicative helicase CMG (cell division control protein 45 homolog (Cdc45)/MCM2–7/Sld5-Psf1-Psf2-Psf3 complex (go-ichi-ni-san; GINS) complex), leading to DNA polymerase loading and the initiation of DNA synthesis.

A crucial protein for completion of a successful DNA replication program is TopBP1, which is required for the firing of DNA replication forks, but is also a key activator of the serine/threonine-protein kinase ATR (ATR) checkpoint kinase [6]. ATR does not participate in the firing of replication origins, but limits unnecessary activation of dormant replication origins and prevents the accumulation of DNA damage during the S phase [7]. How ATR restricts origin firing is not completely understood. Both ATR and TopBP1 are essential proteins required for the survival of proliferating cells [8,9,10]. Mutating the ATR-activation domain (AAD) of TopBP1 resulted in embryonic lethality and cellular senescence in a mouse model [11]. However, it is not well understood why the AAD of TopBP1 is important for cell survival and how it affects the decisions regarding the initiation of DNA replication.

Using a DNA fiber assay and human osteosarcoma (U2OS) cell lines inducibly expressing either wild-type or mutant TopBP1, we investigated how an AAD mutant of TopBP1 affects the initiation of DNA replication during the S phase. We report that the cells expressing an AAD mutant of TopBP1 show increased initiation of DNA replication in the S phase by losing local dormant origin suppression.

## 2. Results

### 2.1. Cells Expressing the TopBP1 AAD Mutant Arrest at G1 and Enter Senescence

To study the role of the ATR-activation domain (AAD) of TopBP1 during an unperturbed cell cycle, we exploited an ATR-inactivating point mutation (W1145R) of TopBP1 [6]. U2OS cells designed to conditionally express the enhanced GFP (eGFP)-TopBP1 wild-type (WT) and the eGFP-TopBP1 W1145R mutant were described in our previous study [12].

**Figure 1:**
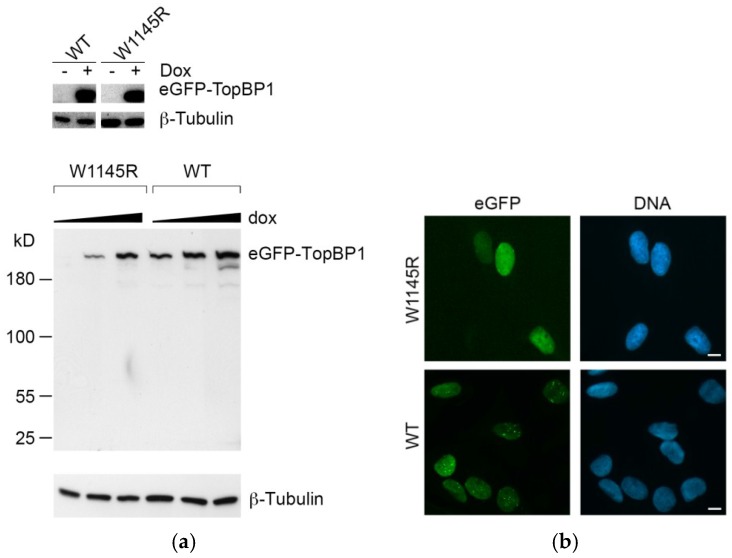
Induced expression of enhanced GFP (eGFP)-DNA topoisomerase 2-binding protein 1 (TopBP1) W1145R and wild-type (WT) cell lines. (**a**) The upper panel shows the TopBP1 signal from non-induced and induced eGFP-TopBP1 WT and W1145R cells (overexposure of the eGFP-TopBP1 and tubulin day 0 and day 1 blots from Figure 2b). In the lower panel, cells were induced to express eGFP-TopBP1 W1145R and WT by an increasing concentration (50, 200, 1000 ng/mL) of doxycycline for 24 h. (**b**) Fluorescence microscopy images of cells expressing either eGFP-TopBP1 W1145R or WT. For WT, cells were induced for 24 h with 200 ng/mL doxycycline, and for W1145R, for 24 h with 1000 ng/mL. The GFP signal is shown in green and Hoechst (DNA) in blue. Scale bar: 10 μm.

Expression was induced by increasing amounts of doxycycline to ascertain that the cell line expressed the eGFP-TopBP1 W1145R mutant as desired. Whole-cell extracts were immunoblotted with an anti-GFP antibody, showing a doxycycline-dependent signal at around 200 kD, resulting from the expression of eGFP-TopBP1 (Figure 1a, upper panel). We did not observe leakage of expression in either cell line when not induced with doxycycline (Figure 1a). For the eGFP-TopBP1 W1145R mutant, we noticed that the level of expression was lower compared to that of the eGFP-TopBP1 WT line. Both eGFP-TopBP1 WT and W1145R localized predominantly in the nucleus (Figure 1b).

**Figure 2:**
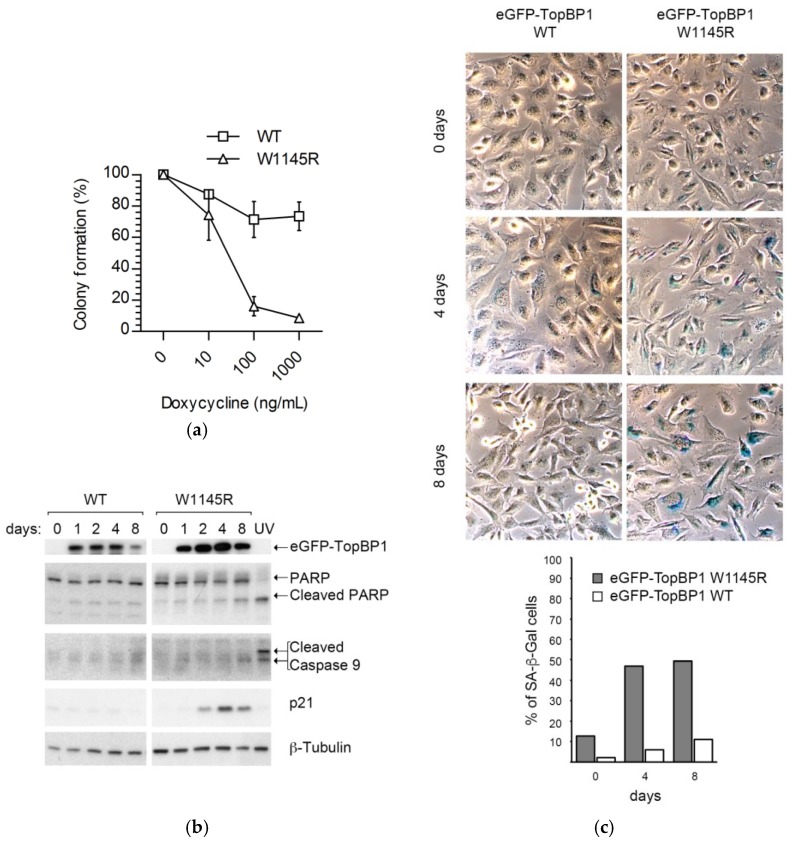
Cells expressing eGFP-TopBP1 W1145R undergo growth arrest and subsequent entry into senescence. (**a**) Colony formation assay of cells expressing eGFP-TopBP1 WT or W1145R. Means of three independent experiments are shown with standard deviations; (**b**) Analysis of apoptotic markers. Whole-cell extracts of cells induced to express either eGFP-TopBP1 W1145R or eGFP-TopBP1 WT for the indicated days were subjected to immunoblot analysis. Non-induced cells treated with 60 J m^−2^ ultraviolet C (254 nm; UV-C) served as a positive control for apoptotic cells (UV); (**c**) The same cells as in panel B were stained for senescence-associated β-galactosidase (SA-β-Gal) activity. At least 170 cells were counted, and the percentage of SA-β-Gal positive cells were scored (bottom panel).

We compared the growth properties of cells expressing eGFP-TopBP1 W1145R to those expressing eGFP-TopBP1 WT in a colony formation assay. Mutant cells growing in the highest doxycycline concentration (1.0 μg/mL) formed only 8% as many colonies compared to those completely excluding doxycycline, while the WT cells formed about 80% colonies (Figure 2a).

The fewer colonies formed by the W1145R TopBP1 mutant cells might reflect cell death or lack of cell division. However, we noted that there was no indication of a loss of viability of the cells plated for the colony assay throughout the one-week experiment. Specifically, to rule out the possibility of apoptosis, we induced the cells to express eGFP-TopBP1 W1145R for 1–8 days and collected attached and floating cells for immunoblot analysis of apoptosis markers. We detected little evidence of apoptosis in these cells, as there was only a marginal elevation of cleaved poly (ADP-ribose) polymerase (PARP) and cleaved caspase-9, while treatment of non-induced cells with 60 J m^−2^ ultraviolet C (254 nm; UV-C) resulted in a robust boost of these apoptosis markers (Figure 2b). These results suggest that the TopBP1 W1145R mutant cells formed fewer colonies due to a decline in cell division. We reasoned that if the cells remain in a viable state without dividing, it could indicate induction of senescence in these cells. Indeed, the increase of cell-cycle inhibitor p21 (Figure 2b) and senescence-associated β-galactosidase (SA-β-Gal; Figure 2c) in cells expressing eGFP-TopBP1 W1145R was evident after as early as four days.

**Figure 3:**
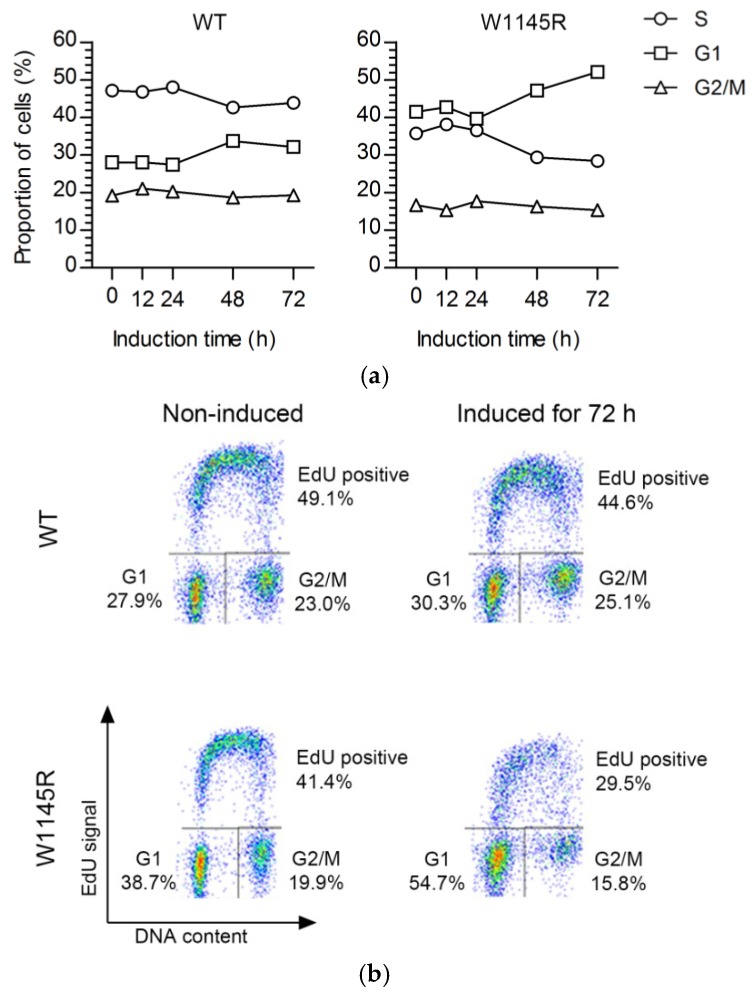
Cells expressing eGFP-TopBP1 W1145R arrest predominantly in gap 1 (G1) phase. (**a**) Flow cytometry analysis of cells. Cells were induced to express eGFP-TopBP1 WT or W1145R for the indicated times, and were pulsed with 5-ethynyl-2′-deoxyuridine (EdU) prior to sample collection to label synthesis (S)-phase cells. (**b**) Flow cytometry profiles of selected samples from panel A.

We next analyzed the cell-cycle progression profiles after 12–72 h of continuous eGFP-TopBP1 W1145R expression. The flow cytometry analysis showed that eGFP-TopBP1 W1145R cells accumulated at the G0/G1 phase with a concomitant reduction of S-phase cells, while the cell-cycle distribution of eGFP-TopBP1 WT cells did not markedly change upon induction (Figure 3a,b). Previously, we showed that cells expressing eGFP-TopBP1 WT continue dividing for several days [12]. We noted that an increasing fraction of cells expressing eGFP-TopBP1 W1145R incorporated less 5-ethynyl-2′-deoxyuridine (EdU) as suggested by the lower, blurry S-phase arc (Figure 3b), indicating problems in DNA replication.

Together, these results show that the cells expressing the TopBP1 AAD mutant remain viable, but a large fraction of cells arrest with a G1-phase DNA content, and, if the expression is prolonged, these cells enter senescence. Since our cells still contain endogenous TopBP1, we conclude that the TopBP1 AAD mutant has a dominant negative effect on cell-cycle progression.

### 2.2. The TopBP1 AAD Mutant Slows Down the DNA Replication Elongation Rate and Increases the Number of Fired Origins

**Figure 4:**
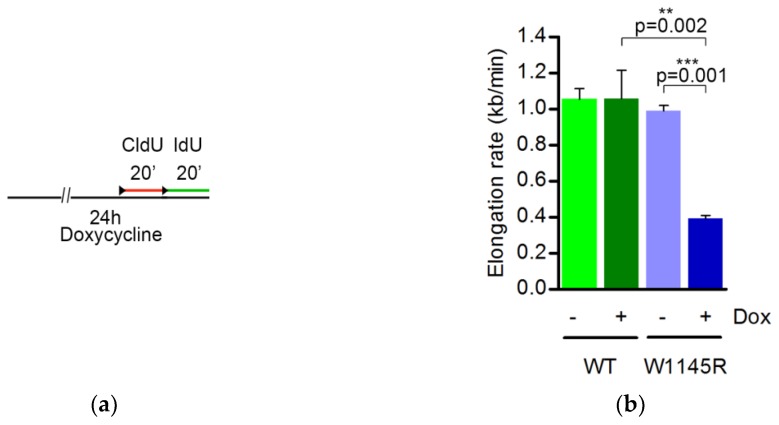

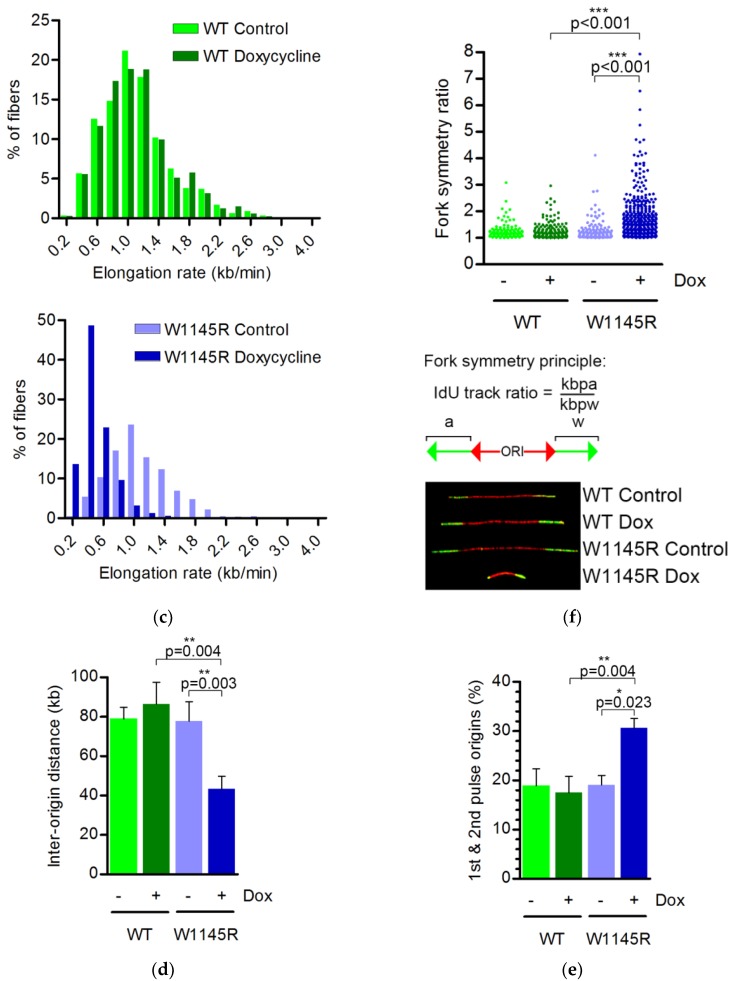
Expression of eGFP-TopBP1 W1145R but not WT causes strong DNA replication stress. (**a**) Scheme for the DNA fiber assay; (**b**) DNA replication fork elongation rate (kb min^−1^); (**c**) The distribution of elongation rate data from panel B; (**d**) Distance between origins (kb); (**e**) Percentage of new origins initiated during both pulses; (**f**) Whisker plot showing fork symmetry ratios in individual fibers. The principle for counting fork symmetry ratios, and examples of representative fibers are shown. For this analysis, we used longer labeling times (45 min instead of 20 min) as it gives longer tracks which are easier to measure. Mean values and standard deviations are shown in panels (**b**,**d**,**e**). The data are from three technical repeats from two independent experiments. For each experiment, 129 to 953 fibers were scored. Statistical significance was calculated using paired samples (when comparing W1145R −Dox vs. +Dox) or unpaired samples (WT +Dox vs. W1145R −Dox) two-tailed Student’s *t*-tests in panels (**b**,**d**,**e**), and a Mann–Whitney test in panel (**f**), using the averages of the individual experiments. *, ** and *** indicate *p* values below 0.05, 0.005 and 0.001, respectively. Representative images of DNA fibers are presented in Figure S1.

The shift in EdU-labeling intensity in cells expressing eGFP-TopBP1 W1145R (Figure 3b) prompted us to concentrate on subsequent analyses of S-phase cells. We performed DNA fiber assays on cells that were induced to express either eGFP-TopBP1 WT or W1145R for 24 h. The cells were sequentially pulse-labeled with 5-chloro-2′-deoxyuridine (CldU) and 5-iodo-2′-deoxyuridine (IdU) for 20 min before analysis (Figure 4a). We observed a dramatic decrease in the average DNA replication elongation rate from 1.0 kb min^−1^ to 0.4 kb min^−1^ (Figure 4b) and a clear shift in the distribution of elongation rates (Figure 4c) when cells were induced to express eGFP-TopBP1 W1145R. Slowed replication fork rates were observed in unperturbed conditions after ATR or serine/threonine-protein kinase Chk1 (Chk1) inhibition or depletion [13,14,15,16], or after Cdc45 overexpression [17]. To further explore if origin firing is increased in mutant TopBP1 cells, we analyzed the inter-origin distance and the fraction of new origins during the combined first and second pulses of labeling. Indeed, the average distance between origins dropped almost by half from 78 to 43 kb (Figure 4d) when expression of eGFP-TopBP1 W1145R was induced, indicating an excessive firing of dormant origins. In line with this, we also observed a significant increase in newly fired origins in cells expressing eGFP-TopBP1 W1145R (Figure 4e). Thus, cells expressing the TopBP1 AAD mutant displayed a severe replication stress phenotype [18]. The strong asymmetry of elongation of fork pairs from the same origin (Figure 4f) suggests that the reduced elongation rate is caused by recurrent stalling of DNA replication. These results strongly suggest that the limitation of (dormant) origin firing is controlled by a TopBP1-mediated pathway that is dependent on a functional AAD. As the excess of WT TopBP1 after doxycycline induction did not affect origin firing or elongation rate, TopBP1 appears not to be rate limiting for DNA replication origin firing.

### 2.3. The TopBP1 AAD Mutant Induces Accumulation of Single-Stranded DNA

**Figure 5:**
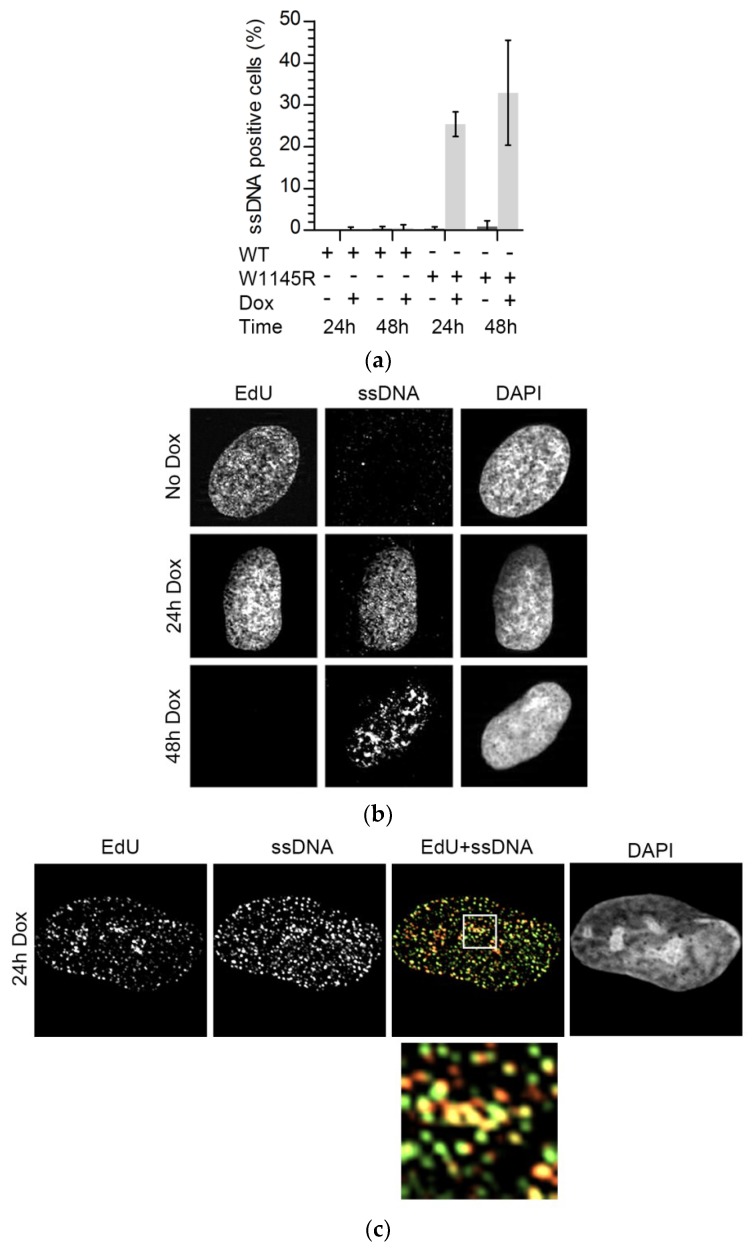

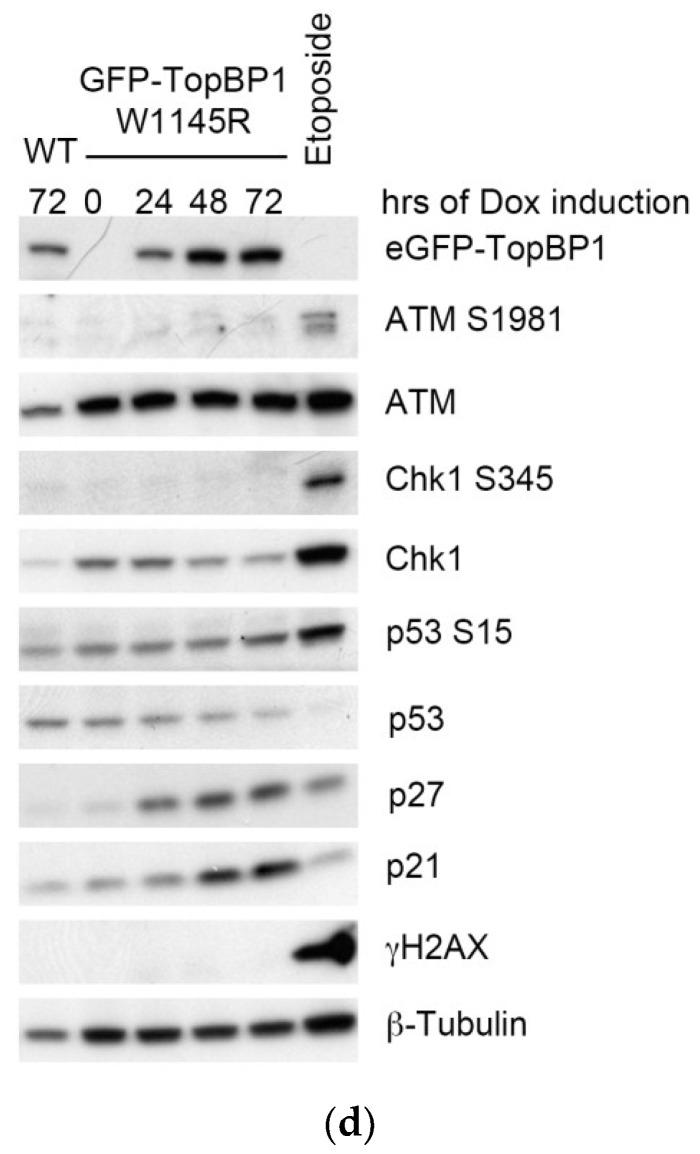
Expression of eGFP-TopBP1 W1145R induces accumulation of single-stranded DNA (ssDNA), but no DNA damage response. (**a**) ssDNA analysis of eGFP-TopBP1 WT and W1145R cells left non-induced or induced for 24 or 48 h. Means of three independent experiments with standard deviations are shown; (**b**) Representative examples of nuclei from panel A. DNA replication foci were labeled with a short pulse of EdU; (**c**) Co-localization of DNA replication foci (yellow) and ssDNA is shown in the overlay image (EdU + ssDNA) and in a magnified region marked by white frame (bottom frame); (**d**) Immunoblot analysis of whole-cell extracts from cells induced to express either eGFP-TopBP1 WT or W1145R for the indicated times. Etoposide was used as a positive control to induce the intra-S-phase damage response (normal human osteosarcoma (U2OS) cells).

ATR or Chk1 inhibition is known to lead to the generation of excess single-stranded DNA (ssDNA) [19,20]. We tested if ssDNA was also present in cells expressing eGFP-TopBP1 W1145R. Indeed, we found that after 24 or 48 h of expression, ssDNA was present in about 30% of the cells, while it was not detected in non-induced or eGFP-TopBP1 WT expressing cells (Figure 5a,b and Figure S2). We noted that ssDNA foci were more enlarged in cells which expressed mutant TopBP1 for 48 h than in cells expressing it for only 24 h. In the latter, the ssDNA foci were more similar to replication foci and overlapped with sites of DNA replication (Figure 5c).

The generation of ssDNA in wild-type cells normally results in the amplification of ATR signaling via the independent recruitment of TopBP1 and ATR to the ssDNA. In our mutant cells, endogenous TopBP1 was still present, which, in principle, could initiate the DNA replication stress response. Indeed, the cells induced to express mutant TopBP1 were still fully capable of activating the Chk1 response when irradiated with UV-C (Figure S3A). Depletion of TopBP1 by two different small interfering RNAs (siRNAs) from the parental U2OS cell strain completely abrogated the Chk1 response to UV-C, showing that the Chk1 response is dependent on TopBP1 in these cells (Figure S3B). To test if the DNA damage response was induced in cells expressing mutant TopBP1, we analyzed the expressions of p21, p27, and phosphorylated serine-protein kinase ATM (ATM), Chk1, p53, and histone H2AX phosphorylated at serine 139 (γH2AX) in immunoblots of whole-cell extracts. While the cells showed elevated levels of p21 and p27, no accumulation of the phosphorylated DNA damage checkpoint markers, ATM S1981, Chk1 S345, p53 S15, or γH2AX was observed (Figure 5d). Increased p21 and p27 protein levels further support the notion of senescence-associated G1 arrest in response to defective TopBP1 signaling rather than an intra-S-phase damage response. Expression of WT TopBP1 did not affect the levels of DNA replication stress markers (Figure 5d).

Taken together, these results show that the failure of TopBP1 signaling during unperturbed DNA replication leads to excess origin firing, decreased replication fork elongation due to excessive fork stalling, and an accumulation of ssDNA. These results resemble the phenotypes of ATR or Chk1-inhibited cells that show excess local origin firing causing defective progression of replication forks [14,20]. Despite a lack of replication checkpoint signaling, the mutant TopBP1 cells did not go into an “intrinsic replication catastrophe” as do cells overexpressing Cdc45 [17].

## 3. Discussion

Using an AAD mutant of TopBP1, we demonstrated that regulation of origin firing is compromised in cells expressing the TopBP1 mutant in the S phase. This observation is in accordance with the essential role of TopBP1 in activating ATR, and with findings that inhibiting the ATR-Chk1 pathway restricts dormant origin firing [14,21,22,23,24]. However, until now, it was not clear if TopBP1 with an inactivating mutation in its ATR-activation domain had a similar effect on replication fork dynamics as occurs after the inhibition of ATR or Chk1. We also report that cells expressing the AAD mutant of TopBP1 accumulate at the G1 phase and enter senescence if expression is prolonged. This is consistent with deletion mutation experiments in mouse, where wild-type TopBP1 was replaced with an AAD mutant [11]. Since our cell lines still had endogenous TopBP1 present, the cell-cycle blocking effect of the AAD mutant TopBP1 is dominant negative, in contrast to the damage response induced by UV-C (Figure S3). Thus, the AAD of TopBP1 appears to be essential for cell-cycle progression, and for the initiation of DNA replication.

DNA replication origins are licensed by loading pre-replication complexes in early G1 well before the restriction point [25]. It is notable that pre-replication complexes are loaded in excess to that which is ultimately used during a given S phase. Most origins remain dormant and are only fired when replication stress leads to problems in fork progression [26]. During stress, dormant origins can be activated to ensure continuation of replication without dramatic slowing down of the whole replication program. However, too many simultaneously active replication forks can be deleterious to cells, leading to replication stress, accumulation of ssDNA, and ultimately, DNA shattering [17,20]. Interestingly, we did not observe initiation of the DNA damage response in cells expressing the mutant TopBP1, despite the severe DNA replication phenotype and accumulation of ssDNA. It is even more surprising considering our observation that the endogenous TopBP1 present in our expression cell lines is capable of inducing Chk1 in response to UV-C (Figure S3). This may be explained by the (replication-dependent) replication protein A (RPA) depletion that we observed after expression of the TopBP1 AAD mutant. It should be noted that the phenotype observed here after expression of TopBP1 AAD is not merely phenocopying the effects of ATR inhibition or depletion. ATR inhibition does not lead to a severe loss of cellular viability in the absence of induced replication stress [15,16,27]. For instance, Jossé et al. [27] did not observe any effect on elongation rates after ATR inhibition, whereas Moiseeva et al. [15] observed reduced elongation rates and increased origin firing, but no fork asymmetry, indicating no augmentation of fork stalling. This is consistent with the role of ATR during replication stress, especially preventing exhaustion of the RPA pool [20]. Here, we observed that expression of the AAD mutant, but not WT TopBP1, induces both replication stress and suppresses ATR-dependent damage responses. Whereas the latter could be a secondary effect of suppressing ATR activation by the AAD mutant, the phenotype is more consistent with a dual role of TopBP1 coupling the initiation of DNA replication with the suppression of neighboring origins.

Moreover, several recent studies identified Ewing’s tumor-associated antigen 1 (ETAA1) as a second ATR activator [28,29,30,31]. It was noted previously that U2OS cells have very low ETAA1 protein levels [30]. This could explain the lack of replication stress-induced Chk1 phosphorylation, as well as the severity of the phenotype of the AAD mutant expression observed in this study. ETAA1 activates ATR in response to DNA replication stress independently of TopBP1, but is not implicated to participate in DNA replication initiation or elongation.

It was proposed previously that a fired origin suppresses the firing of nearby origins within a replication cluster. This negative origin interference was first found in yeast [32,33] and later applied to human cells as well [34]. How negative origin interference functions mechanistically is not understood. Two models can be envisioned that are not necessarily mutually exclusive.

The more efficiently firing origin could deplete replication factors resulting in differential firing efficiency in a local cluster of origins. The less efficient origin in the cluster would thereafter be inactivated due to its replication by a traversing fork from the nearby more efficient origin. Alternatively, there could be a factor or factors that inhibit nearby origin activation in the vicinity of a fired origin.

TopBP1 was not previously observed to be required for the elongation of DNA replication [35,36,37], which would explain the increased stalling of forks we observe. It is possible, however, that fork stalling is a naturally frequent event in cells, which would require continuous TopBP1-ATR for re-initiation.

**Figure 6:**
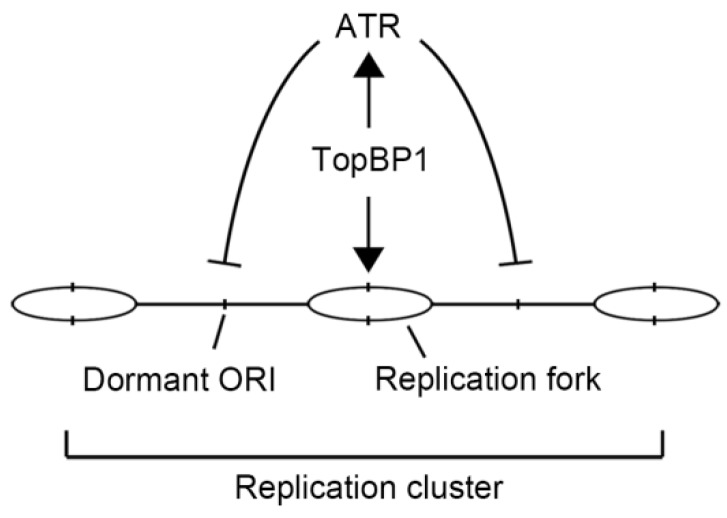
A model for the role of TopBP1 in the S phase. During the S phase, TopBP1 activates the firing of new forks, and the TopBP1 local activation of serine/threonine-protein kinase ATR (ATR) inhibits the firing of nearby dormant origins.

Another possibility that is consistent with the negative origin interference is that, once TopBP1 binds to the pre-initiation complex and activates an origin, it simultaneously induces local activation of the ATR pathway leading to an inhibition of nearby origin activation (see Figure 6). Such an essential function of TopBP1 would explain the ostensibly contradictory roles in activating and suppressing origin firing.

## 4. Materials and Methods

### 4.1. Cell Culture

The preparation and cultivation of eGFP-TopBP1 WT and W1145R cells were described previously [12]. All cell lines were regularly checked for contamination. The SA-β-Gal assay was performed according to the manufacturer’s instructions (Millipore).

### 4.2. Colony Formation Assay

Approximately 5000 cells were plated per 10-cm dish and then grown with or without doxycycline for eight days until colonies of over 50 cells appeared in the no-doxycycline control plates. Colonies were fixed with methanol and stained with 0.5% crystal violet in 25% methanol. The number of colonies was counted with a custom automated analysis using CellProfiler (r11710) [38].

### 4.3. Flow Cytometry

Cells were labeled with 10 µM EdU for 15 min before harvesting. Harvested cells were washed with 1% bovine serum albumin/phosphate-buffered saline (BSA/PBS) and fixed with 2% paraformaldehyde for 15 min. EdU detection (Click-iT EdU Alexa Fluor 647) was done according to the manufacturer’s instructions (Life Technologies, Carlsbad, CA, USA). DNA was stained with 4′,6-diamidino-2-phenylindole (DAPI; Sigma-Aldrich, St. Louis, MI, USA). Flow cytometry data were acquired with a FACSCanto machine using the FACSDiva software (BD Biosciences, Franklin Lakes, NJ, USA). Data analysis was done using the FlowJo software (FlowJo, LLC, Ashland, OR, USA).

### 4.4. DNA Fiber Assays

For the DNA fiber assays, cells were either non-induced or induced to express eGFP-TopBP1 WT or W1145R for 24 h before supplementing the medium with 25 μM IdU (Sigma-Aldrich) for 20 min (first labeling). The cells were then washed, and 250 μM CldU (Sigma-Aldrich) was added to fresh medium for 20 min (second labeling). Preparation of DNA spreads was done as described previously [17].

### 4.5. Single-Stranded DNA (ssDNA) Analysis and Immunofluorescence Microscopy

DNA was labeled with 10 µM 5-bromo-2′-deoxyuridine (BrdU) for 36 h. Sample preparation was performed as described previously [17]. To detect incorporated BrdU only in regions of ssDNA, the DNA was directly immunolabelled (without denaturation) with a primary monoclonal antibody (1:1500 dilution) of rat-anti-BrdU (Clone BU1/75 ABD Serotec) and a secondary anti-rat Alexa Fluor 555 conjugate. Total DNA was counterstained with DAPI. Fluorescence images were acquired using a Zeiss Axio Imager.Z1 at 630-fold magnification.

For the fluorescence microscopy in Figure 1b, cells were prepared as described previously [12]. Briefly, cells were fixed with 3% paraformaldehyde, permeabilized with 0.2% Triton X-100-PBS, and stained for DNA with Hoechst 33258. Wide-field fluorescent images were obtained with Axiocam HR color using a Zeiss Axioplan 2 microscope with a 40× Zeiss Plan-Neofluar objective.

### 4.6. Immunoblotting

Immunoblotting was performed as described previously [12]. Primary antibodies (1:1000 dilutions unless otherwise stated) were from Cell Signaling (α-GFP D5.1 (1:2000), α-PARP #9542, α-caspase 9 #9502 (1:2000), α-ATM D2E2, α-ATM phospho-S1981 10H11.E12, α-Chk1 phospho-S345 133D3, α-p53 phospho-S15 16G8 (1:2000), α-p27 D69C12, and α-phospho-S780 pRB #9307), Millipore (α-p21 #05-345, α-β-Tubulin KMX-1 (1:20000), and α-γH2AX JBW301), and Santa Cruz Biothechnology (α-p53 sc-6243 (1:400), α-Chk1 sc-8408, and α-Cyclin A sc-751). Secondary antibodies (1:40000 dilution) for immunoblotting were peroxidase-conjugated goat α-rabbit or α-mouse immunoglobulin G (IgG; Jackson Immunoresearch Laboratories).

## Supplementary Materials

Supplementary materials can be found at http://www.mdpi.com/1422-0067/19/8/2376/s1.

## Abbreviations

AAD	ATR activating domain (in TopBP1)
ATM	Serine-protein kinase ATM
ATR	Serine/threonine-protein kinase ATR
BSA	Bovine serum albumin
Cdc45	Cell division control protein 45 homolog
Cdc6	Cell division control protein 6 homolog
Cdt1	DNA replication factor Cdt1
Chk1	Serine/threonine-protein kinase Chk1
CldU	5-Chloro-2′-deoxyuridine
CMG	Cdc45-MCM-GINS complex
DDK	Dbf4-dependent kinase
EdU	5-Ethynyl-2′-deoxyuridine
ETAA1	Ewing’s tumor-associated antigen 1
eGFP	Enhanced green fluorescent protein
GEMC1	Geminin coiled-coil domain-containing protein 1
GINS	Sld5-Psf1-Psf2-Psf3 complex (go-ichi-ni-san)
H2A.X/γH2AX	Phosphorylated histone H2AX
IdU	5-iodo-2′-deoxyuridine
kb	kilobase
MCM2-7	DNA replication licensing factor MCM2-7
ORI	Origin of DNA replication
p21	Cyclin-dependent kinase inhibitor 1
p27	Cyclin-dependent kinase inhibitor 1B
p53	Cellular tumor antigen p53
PARP	Poly (ADP-ribose) polymerase
PBS	Phosphate-buffered saline
RecQL4	ATP-dependent DNA helicase Q4
RPA	Replication protein A complex
S-CDK	S-phase-specific cyclin dependent kinase
SA-β-Gal	Senescence-associated beta-galactosidase
ssDNA	Single-stranded DNA
TopBP1	DNA topoisomerase 2-binding protein 1
U2OS	Human osteosarcoma cell line
UV-C	Ultraviolet C (254 nm)
WT	Wild type
