# Supplementary figures and images for: The ATR-Activation Domain of TopBP1 Is Required for the Suppression of Origin Firing during the S Phase
Source: Int J Mol Sci. 2018 Aug 13;19(8):2376. doi: 10.3390/ijms19082376 (PMC6121618; doi:10.3390/ijms19082376)

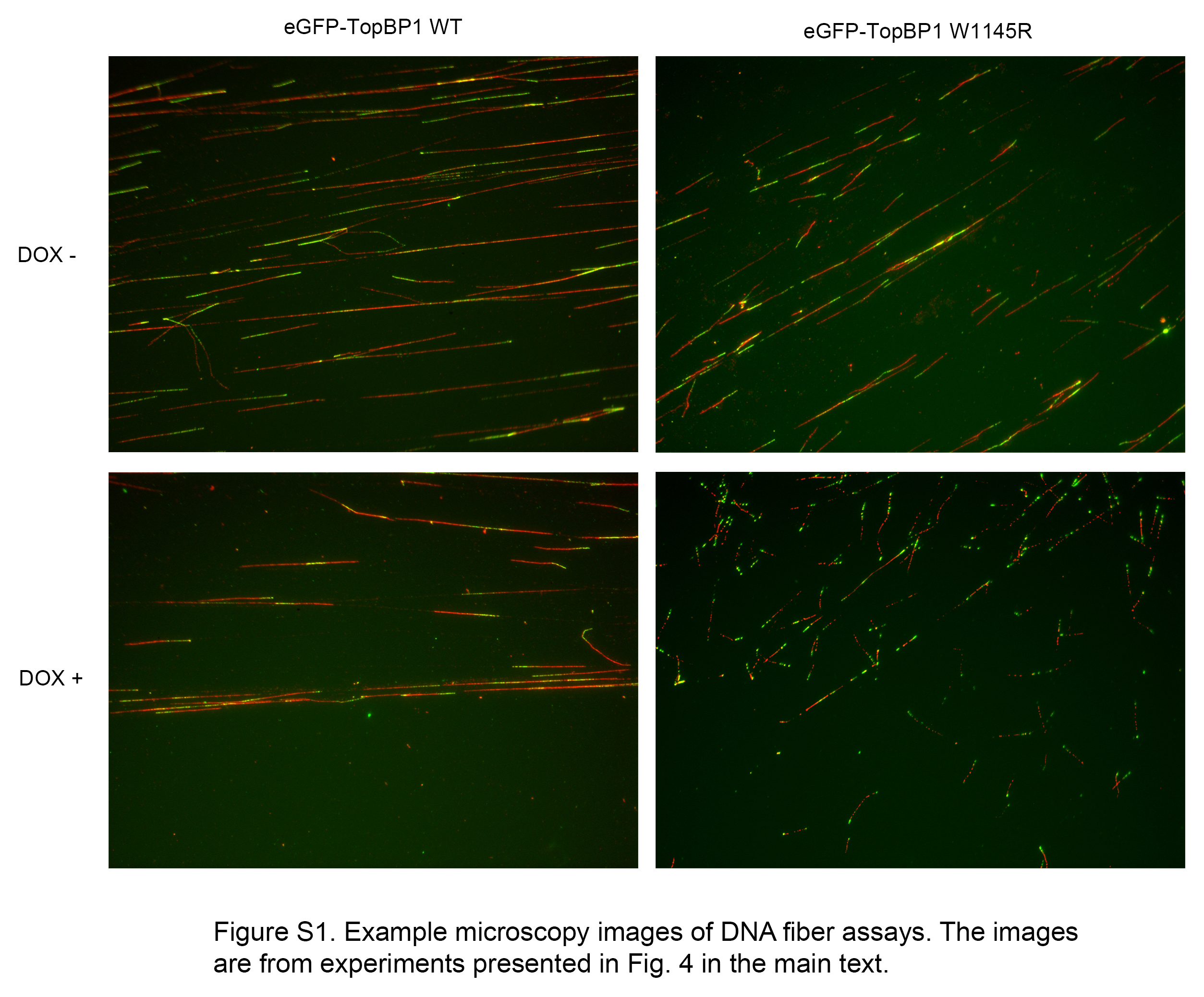

Supplement: Supplementary file 1 [file ijms-19-02376-s001.zip › ijms-339234-final si/FigS1.jpg]

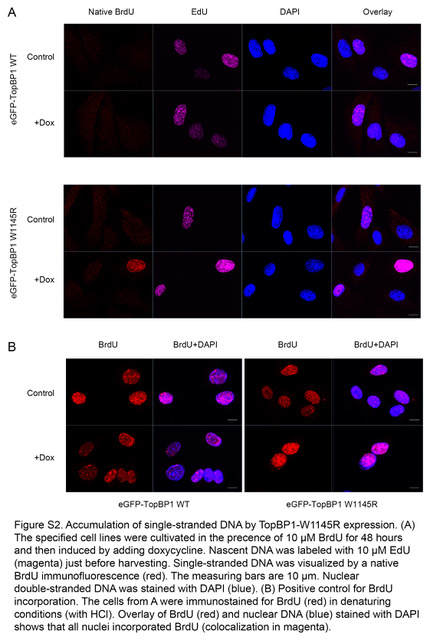

Supplement: Supplementary file 1 [file ijms-19-02376-s001.zip › ijms-339234-final si/FigS2.jpg]

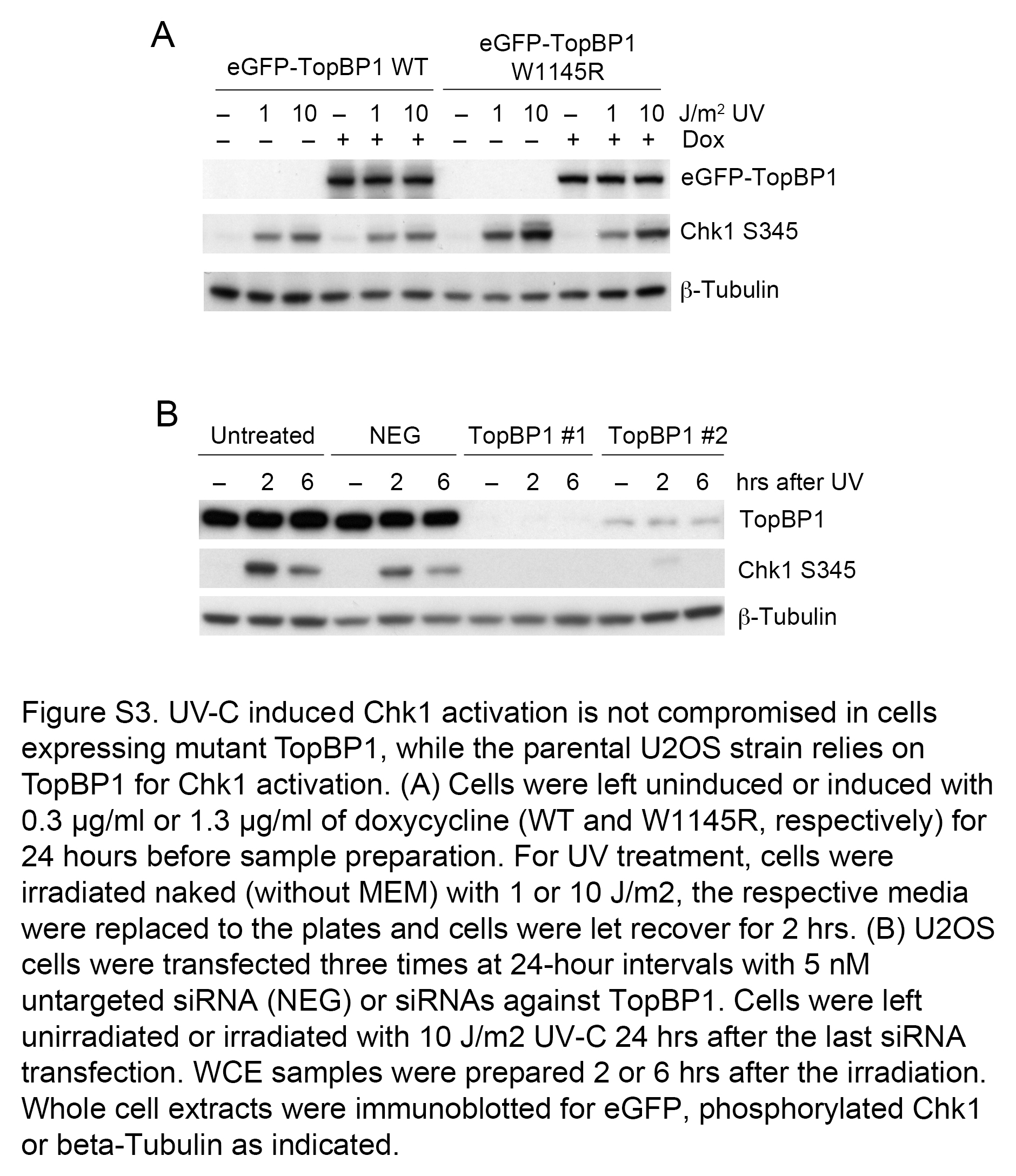

Supplement: Supplementary file 1 [file ijms-19-02376-s001.zip › ijms-339234-final si/FigS3.jpg]
